# Long-Term SARS-CoV-2 Specific Immunity Is Affected by the Severity of Initial COVID-19 and Patient Age

**DOI:** 10.3390/jcm10194606

**Published:** 2021-10-08

**Authors:** Margarethe Konik, Monika Lindemann, Markus Zettler, Lara Meller, Sebastian Dolff, Vera Rebmann, Peter A. Horn, Ulf Dittmer, Adalbert Krawczyk, Leonie Schipper, Mirko Trilling, Olympia Evdoxia Anastasiou, Sina Schwarzkopf, Laura Thümmler, Christian Taube, Christoph Schöbel, Thorsten Brenner, Eva-Maria Skoda, Benjamin Wilde, Anja Gäckler, Oliver Witzke, Hana Rohn

**Affiliations:** 1 Department of Infectious Diseases, West German Centre of Infectious Diseases, University Hospital Essen, University Duisburg-Essen, 45147 Essen, Germany; margarethe.konik@uk-essen.de (M.K.); markus.zettler@uk-essen.de (M.Z.); lara.meller@uk-essen.de (L.M.); sebastian.dolff@uk-essen.de (S.D.); adalbert.krawczyk@uk-essen.de (A.K.); leonie.schipper@uk-essen.de (L.S.); oliver.witzke@uk-essen.de (O.W.); 2 Institute for Transfusion Medicine, University Hospital Essen, University Duisburg-Essen, 45147 Essen, Germany; monika.lindemann@uk-essen.de (M.L.); vera.rebmann@uk-essen.de (V.R.); Peter.Horn@uk-essen.de (P.A.H.); sina.schwarzkopf@uk-essen.de (S.S.); laura.thuemmler@uk-essen.de (L.T.); 3 Institute for Virology, University Hospital Essen, University Duisburg-Essen, 45147 Essen, Germany; ulf.dittmer@uk-essen.de (U.D.); mirko.trilling@uk-essen.de (M.T.); olympiaevdoxia.anastasiou@uk-essen.de (O.E.A.); 4 Department of Pneumology, University Medicine Essen-Ruhrlandklinik, University Duisburg-Essen, 45147 Essen, Germany; christian.taube@rlk.uk-essen.de (C.T.); christoph.schoebel@rlk.uk-essen.de (C.S.); 5 Department of Anesthesiology and Intensive Care Medicine, University Hospital Essen, University Duisburg-Essen, 45147 Essen, Germany; Thorsten.brenner@uk-essen.de; 6 Clinic for Psychosomatic Medicine and Psychotherapy, LVR University Hospital, University Hospital Essen, University Duisburg-Essen, 45147 Essen, Germany; eva.skoda@uk-essen.de; 7 Department of Nephrology, University Hospital Essen, University Duisburg-Essen, 45147 Essen, Germany; benjamin.wilde@uk-essen.de (B.W.); anja.gaeckler@uk-essen.de (A.G.)

**Keywords:** COVID-19, SARS-CoV-2, immunity, age, T cell, SARS-CoV-2 IgG, spike protein, Membrane protein

## Abstract

The coronavirus disease 2019 (COVID-19) caused by the severe acute respiratory syndrome coronavirus 2 (SARS-CoV-2) is currently the greatest medical challenge. Although crucial to the future management of the pandemic, the factors affecting the persistence of long-term SARS-CoV-2 immunity are not well understood. Therefore, we determined the extent of important correlates of SARS-CoV-2 specific protection in 200 unvaccinated convalescents after COVID-19. To investigate the effective memory response against the virus, SARS-CoV-2 specific T cell and humoral immunity (including virus-neutralizing antibodies) was determined over a period of one to eleven months. SARS-CoV-2 specific immune responses were present in 90% of individual patients. Notably, immunosuppressed patients did not have long-term SARS-CoV-2 specific T cell immunity. In our cohort, the severity of the initial illness influenced SARS-CoV-2 specific T cell immune responses and patients’ humoral immune responses to Spike (S) protein over the long-term, whereas the patients’ age influenced Membrane (M) protein-specific T cell responses. Thus, our study not only demonstrated the long-term persistence of SARS-CoV-2 specific immunity, it also determined COVID-19 severity and patient age as significant factors affecting long-term immunity.

## 1. Introduction

The first diagnosis of coronavirus disease 2019 (COVID-19) cases caused by the severe acute respiratory syndrome coronavirus-type 2 (SARS-CoV-2) occurred in Wuhan, China, in December 2019, and its subsequent spread starting in spring 2020 marked the beginning of a challenging global pandemic and public health crisis, which is unprecedented in modern history [1]. COVID-19 comprises a wide spectrum of clinical manifestations, ranging from moderate courses of infection with very mild symptoms to life-threatening viral pneumonia. The latter is more frequent in patients with preexisting conditions such as compromised immunity, and the elderly [2]. SARS-CoV-2 infections do not necessarily cause COVID-19 and a relevant fraction (in some studies up to almost half of all infections) occur without symptoms [3,4]. Despite the recent development of highly effective vaccines, the understanding of sustained SARS-CoV-2 immunity in the convalescent unvaccinated population remains crucial for future decision making regarding the ongoing pandemic [5,6]. It is particularly relevant for countries with lower vaccine coverage. There is concern that waning immunity may eventually fail to prevent all re-infection. Others, and we, have studied the antibody response against the virus and its kinetics in various population groups [3,7,8,9,10]. Several of these longitudinal studies report a biphasic decrease in both the levels of SARS-CoV-2 specific antibodies (IgA, IgG and neutralizing antibodies) with a plateau maintained over several months. Immunological memory depends not only on maintaining high antibody titers but also on the generation of specific T cells that immediately respond if the host reencounters the virus [11]. In several countries, convalescent people are considered to be protected against reinfections and are treated similarly to vaccinated individuals with regard to exemptions from public health restrictions operative for non-protected people. This poses questions such as the following: How long will the protection last, particularly in asymptomatic or mildly ill individuals? Does the severity of symptoms influence cellular and humoral immune responses and their sustainability? Which factors influence the persistence of long-term immune responses? To address these questions, the cellular and humoral immune response was assessed in a total of 200 unvaccinated SARS-CoV-2 convalescents. Patients were recruited from the post-COVID-19 outpatient clinic of the Department of Infectious Diseases at the University Hospital Essen, since May 2020.

## 2. Materials and Methods

### 2.1. Study Subjects and Sampling

This cross-sectional, prospective study included SARS-CoV-2 unvaccinated convalescent individuals who presented to the post-COVID-19 outpatient clinic of the Department of Infectious Diseases of the University Hospital Essen in May 2020. To reach as many convalescents as possible, we drew attention to the study by the Health Department of the city of Essen and via the public media. Inclusion criteria were an age at least 18 years and previously confirmed detection of SARS-CoV-2 viral RNA in swab or sputum by reverse transcription polymerase chain reaction (RT-PCR); exclusion criterion was vaccination against SARS-CoV-2. Patients were grouped into categories according to their worst disease manifestation at the time of primary COVID-19, as defined by World Health Organization (WHO) criteria (asymptomatic or mild disease, i.e., WHO score 1–2; hospitalized with moderate disease, i.e., WHO score 3–4, severe disease requiring intensive care treatment, i.e., WHO score 5–7). Peripheral blood samples were collected from all patients for immunological analyses. The median interval between the PCR-confirmed SARS-CoV-2 infection and the presentation to the post-COVID-19 outpatient clinic was 5 months (range 1–11 months). The study was approved by the local ethics committee (approval no. 20-9374-BO) and was performed in accordance with ethics standards noted in the 1964 Declaration of Helsinki and its later amendments or comparable ethics standards. All patients provided informed consent for participation in the study.

### 2.2. T Cell ELISpot Assays for S- and M-Derived SARS-CoV-2 Peptides

To explore the SARS-CoV-2 specific T cell response, we collected peripheral blood mononuclear cells (PBMCs) for interferon gamma (IFN-γ) enzyme-linked immunospot (ELISpot) assays. For 197 of 200 patients, ELISpot assays were performed applying recently described methodologies [11,12]. To cover a broad range of putative antigens for Spike (S) protein and Membrane (M) protein and to increase sensitivity, the following four peptide pools or proteins were used: (I) PepTivator SARS-CoV-2 protein S1/S2 (Miltenyi Biotec), (II) PepTivator SARS-CoV-2 protein S1 protein (Miltenyi Biotec), (III) an S1 protein antigen of SARS-CoV-2 (Sino Biological) or (IV) PepTivator SARS-CoV-2 membrane (M) protein (Miltenyi Biotec). The peptide mix (PepTivator) of the S1/S2 protein covers the immunodominant domains, whereas the peptide mix corresponding to the M protein covers the complete sequence of the glycoprotein. The peptide pools consist mainly of 15-mer sequences with an overlap of 11 amino acids. Per cell culture, 250,000 PBMCs were tested, and IFN-γ production was measured after 19 h, as previously described [11,13]. Spot numbers were evaluated by an ELISpot reader (AID Fluorospot; Autoimmun Diagnostika GmbH, Strassberg, Germany). Mean values of duplicate cell cultures were considered. SARS-CoV-2 specific spots were determined as stimulated minus non-stimulated (background) values (spot increment). A positive response was defined as a threefold increase in SARS-CoV-2 specific spots compared with background and at least three spots above background. This cut-off was set on the basis of the negative control values, as previously described. The ELISpot results were evaluated both for all S peptide pool/protein antigens and M peptide pools individually and in aggregated form (S plus M ELISpot results).

### 2.3. SARS-CoV-2 Specific Antibody Detection

SARS-CoV-2 specific antibodies were detected by two separate methods. Initially, for 84 patients, SARS-CoV-2 immunoglobulin (IgG) against the spike glycoprotein were detected by a Communauté Européenne (CE)-marked anti-SARS-CoV-2 IgG semi-quantitative enzyme-linked immunosorbent assay (ELISA) (Euroimmun, Luebeck, Germany) in accordance with the manufacturer’s instructions. The ELISA plates were coated with recombinant SARS-CoV-2 spike protein (S1 domain). Serum samples were automatically analyzed at a dilution of 1:101 with the Immunomat (Virion\Serion). The results were given as the ratio of patient sample to control sample. An antibody ratio higher than 1.1 was considered positive, a ratio of 0.8 to less than 1.1 was considered borderline, and a ratio lower than 0.8 was considered negative. According to the manufacturer’s instructions, an evaluation of a borderline result is not possible. For statistical analysis, only the clearly positive or negative results of these semi-quantitative analyses were taken into account and dichotomized with respect to the presence of SARS-CoV-2 IgG antibodies.

After a quantitative automatized anti-SARS-CoV-2 IgG chemiluminescent enzyme immunoassay (CLIA) (LIAISON SARS-CoV-2 TrimericS IgG assay, Diasorin, Saluggia, Italy) became available in February 2021, the detection method for SARS-CoV-2 IgG antibodies was changed, and 128 patients were assessed using this method (with 17 patients tested with both antibody detection methods). According to the manufacturer’s recommendations for CLIA, an arbitrary unit per milliliter (AU/mL) ratio of less than 13.0 was considered negative and a ratio of 13.0 or higher was considered positive. A conversion of AU/mL to binding antibody units (BAU/mL), which correlate with the WHO standard, is possible with the following equation: BAU/mL = 2.6*AU/mL. The upper limit of quantification of the CLIA test without dilution is 800.0 AU/mL (2080 BAU/mL).

### 2.4. Cells and Virus

Vero E6 cells were purchased from ATCC (Manassas, VA, USA; ATCC^®^ RL-1586™) and maintained in Dulbecco’s modified Eagle’s medium (DMEM) supplemented with 1% fetal calf serum (FCS), penicillin (100 IU/mL), and streptomycin (100 μg/mL). SARS-CoV-2 was isolated from a nasopharyngeal swab of a patient suffering from COVID-19 in April 2020 as previously described [14]. The virus was propagated on Vero E6 cells cultured in DMEM containing 10% (*v*/*v*) FCS and supplemented with penicillin (100 IU/mL), streptomycin (100 μg/mL), ciprofloxacin (10 μg/mL), and amphotericin B (2.5 μg/mL). Viral titers were determined by endpoint dilution assay and expressed as 50% tissue culture infective dose (TCID50)/mL.

### 2.5. Virus Neutralization Assay

The neutralizing capability of antibodies against SARS-CoV-2 in patient serum was quantified using previously described methods [11]. Serial dilutions (1:20–1:2560) of serum samples were incubated with 100 TCID50 of SARS-CoV-2 for 1 h at 37 °C and subsequently added to confluent Vero E6 cells cultured in 96-well microtiter plates. On day 2 after infection, cells were stained with crystal violet (Roth, Karlsruhe, Germany) and analyzed for the appearance of virus-induced cytopathic effects (CPE) by light microscopy. The neutralizing titer was defined as the reciprocal of the highest serum dilution at which no CPE breakthrough was observed in any of the triplicate cultures. SARS-CoV-2 specific neutralizing antibodies were evaluated in 176 out of 200 patients.

### 2.6. Statistics and Data Analysis

Statistical analyses were performed with IBM SPSS-Statistic 23 (SPSS Inc., Chicago, IL, USA) and GraphPad Prism 9 (GraphPad Software, San Diego, CA, USA) software. Data sets were analyzed with the Mann–Whitney *U*-test, the Kruskal–Wallis test, the Friedman test, or Spearman’s rank-order correlation tests. Univariate and multivariate linear regression models were used to estimate relationship between the independent variables. Two-sided *p*-values lower than 0.05 were considered significant.

## 3. Results

### 3.1. Participants

This prospective cross-sectional study enrolled a total of 200 SARS-CoV-2 PCR-confirmed unvaccinated convalescent patients presenting to the post-COVID-19 outpatient clinic of the Department of Infectious Diseases of the University Hospital Essen. Cohort characteristics are displayed in Table 1.

**Figure 1:**
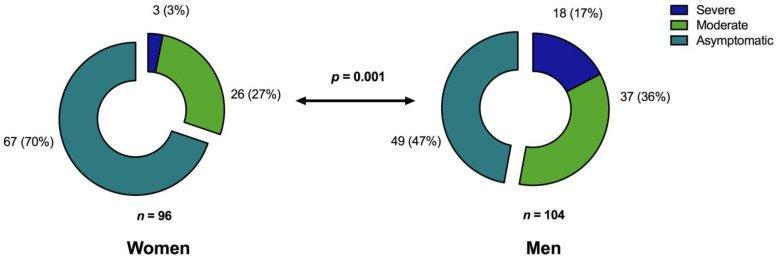
Proportion of COVID-19 severity levels in relation to patient sex [*n* (%)]. Chi-square (*χ*2) test for trend indicated that men experience significantly more severe COVID-19 than women (*p* = 0.001) according to the clinical classification of severity.

Among all patients, 104 (52%) were men with a median age of 50 years (range, 19–87 years). Regarding the severity of initial COVID-19, 84 patients (42%; 55 men; median age, 57 years; range 24–87 years) required hospitalization, and 21 of these (10.5%; 18 men; median age, 56 years; range 25–77 years) had a more severe COVID-19 course requiring intensive care treatment (WHO Score 5–6; Figure 1).

The group of hospitalized patients included significantly more men than women (*p =* 0.001). Hospitalized patients with moderate or severe COVID-19 were significantly older compared with patients with asymptomatic or mild disease (*p <* 0.0001, Table 1).

### 3.2. Simultaneous T Cell ELISpot Assays for S and M SARS-CoV-2 Peptides Increase the Probability of Detecting T Cell Immunity

**Figure 2:**
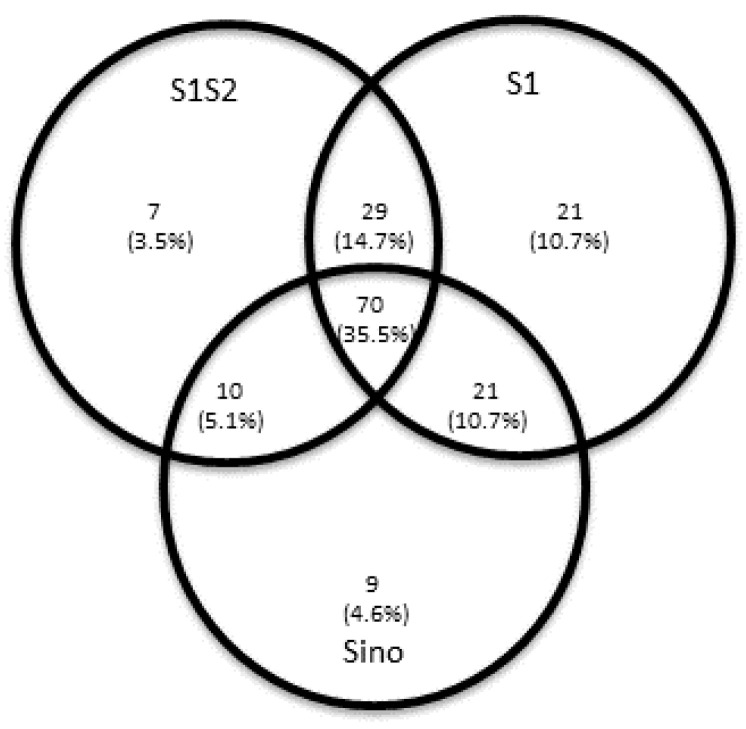
Venn diagram of convalescent patients [*n* (%)] with detectable SARS-CoV-2 reactive T cells as determined by enzyme-linked immunospot (ELISpot) analysis after stimulation with the various Spike (S)-peptide pools. The S protein is responsible for entry into the host cell and consists of two subunits: The S1 subunit contains the receptor-binding domain, which binds to the host cell receptor; the S2 subunit subsequently mediates the fusion of the viral envelope and cell membrane. No SARS-CoV-2 specific T cell response to any S peptide/protein antigen used was detectable in 30 patients.

IFNγ ELISpot analyses were used to determine the magnitude of SARS-CoV-2 specific T cell responses to the SARS-CoV-2 Spike (S) and Membrane (M) proteins. SARS-CoV-2 IFNγ ELISpot assays revealed in 60 out of 197 patients (30%) no demonstrable T cellular response to the M-peptide pool, and in 30 out of 197 patients (15.2%) no demonstrable cellular response to any of the used S-peptide pools. A variance in detectable T cell immunity was found among the various S-peptide pools used (Figure 2).

**Figure 3:**
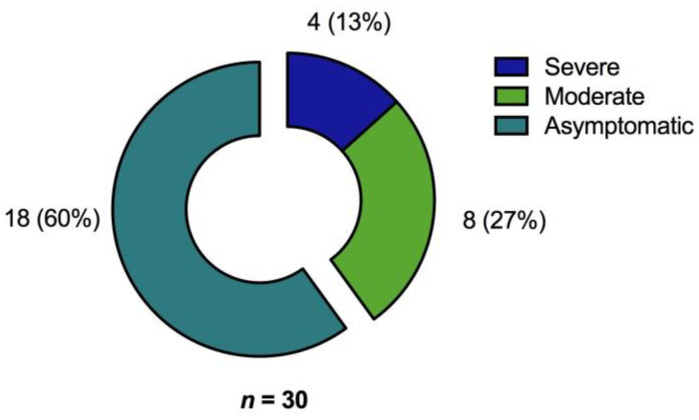
Proportion of convalescent patients with no detectable SARS-CoV-2 reactive T cells as determined by ELISpot analysis (*n* = 30) after stimulation with the various S-peptide pools with regard to primary COVID-19 severity [*n* (%)].

Only 70 out of 197 patients (35.5%) had a T cell specific response to all three different SARS-CoV-2 S peptide pool/protein antigens used in the ELISpot assays. Thus, an examination of only a single peptide pool would have underestimated T cell responses in the majority of patients. Most of those patients with no T cell response in the ELISpot assay belonged to the asymptomatic patient group (Figure 3).

Notably, we found a statistically significant correlation between the magnitude of various T cell IFNγ ELISpot responses to the three separate S-peptide pools and the M-peptide pools (*p* < 0.0001). The total population of peripheral B and T cells was not affected by the primary severity of COVID-19 among convalescent patients, and no association was observed between the total number of CD4^+^ or CD8^+^ T cells and T-cell reactivity as determined by ELISpot analysis for S and M peptides.

#### 3.2.1. Immunosuppression Is an Independent Risk Factor for Lack of Response to SARS-CoV-2 Antigen-Specific Stimulation in ELISpot Analyses

**Figure 4:**
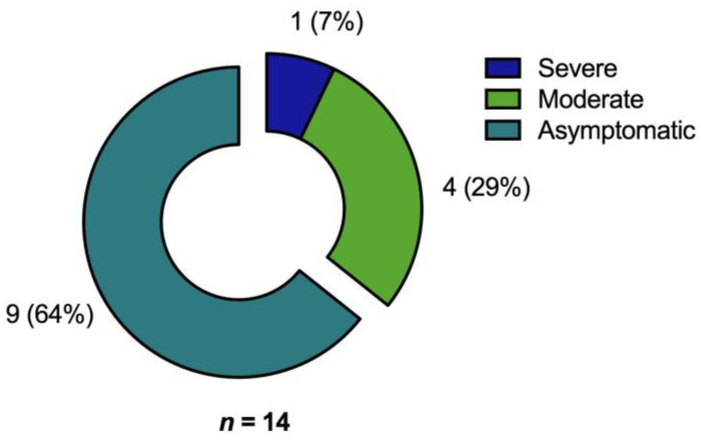
Proportion of convalescent patients with no detectable SARS-CoV-2 reactive T cells as determined by ELISpot analysis (*n* = 14) after stimulation with the various S and M peptide pools with regard to primary COVID-19 severity [*n* (%)].

For 14 patients (7%; patients per group: asymptomatic or mild disease, *n* = 9; moderate disease, *n* = 4; severe disease, *n* = 1, Figure 4) no response to any (S and M protein) of the tested SARS-CoV-2 proteins was detected by the ELISpot assay.

Patients with a compromised immune system are often unable to generate an adequate immune response and are therefore considered particularly at risk for COVID-19. A total of 6 patients received immune suppressing medication and were therefore considered immunocompromised (*n* = 2 after solid organ transplantation, *n* = 1 after allogeneic stem cell transplantation, *n* = 1 for multiple sclerosis, *n* = 1 for COPD and *n* = 1 for Crohn’s disease). Univariate analysis determined that this drug-induced immunosuppressive condition (*p* = 0.029; hazard ratio [HR], 7.42; 95% confidence interval [CI], 1.23–44.65), and existence of anti-SARS-CoV-2 antibody response (*p* < 0.0001; HR, 0.056; 95%CI, 0.017–0.186) was significantly associated with the absence or presence of a specific T cell response towards Membrane (M) or Spike (S) proteins. Concordantly, a multivariate analysis confirmed that these two variables were independently linked to the presence of a T cell specific response against SARS-CoV-2 after primary infection (immunosuppression, *p* = 0.025; HR, 12.53; 95%CI, 1.38–113.95; presence of anti-SARS-CoV-2 antibodies, *p* < 0.0001; HR, 0.052; 95%CI, 0.014–0.183; Table 2). In our cohort, there was no association between severity of initial COVID-19, time since primary infection, patient sex, or age, and the absence of T cell responses as determined by ELISpot assay.

#### 3.2.2. The Magnitude of SARS-CoV-2 Specific T Cell Response Is Dependent on the Severity of Primary COVID-19 and Patient Age

**Figure 5:**
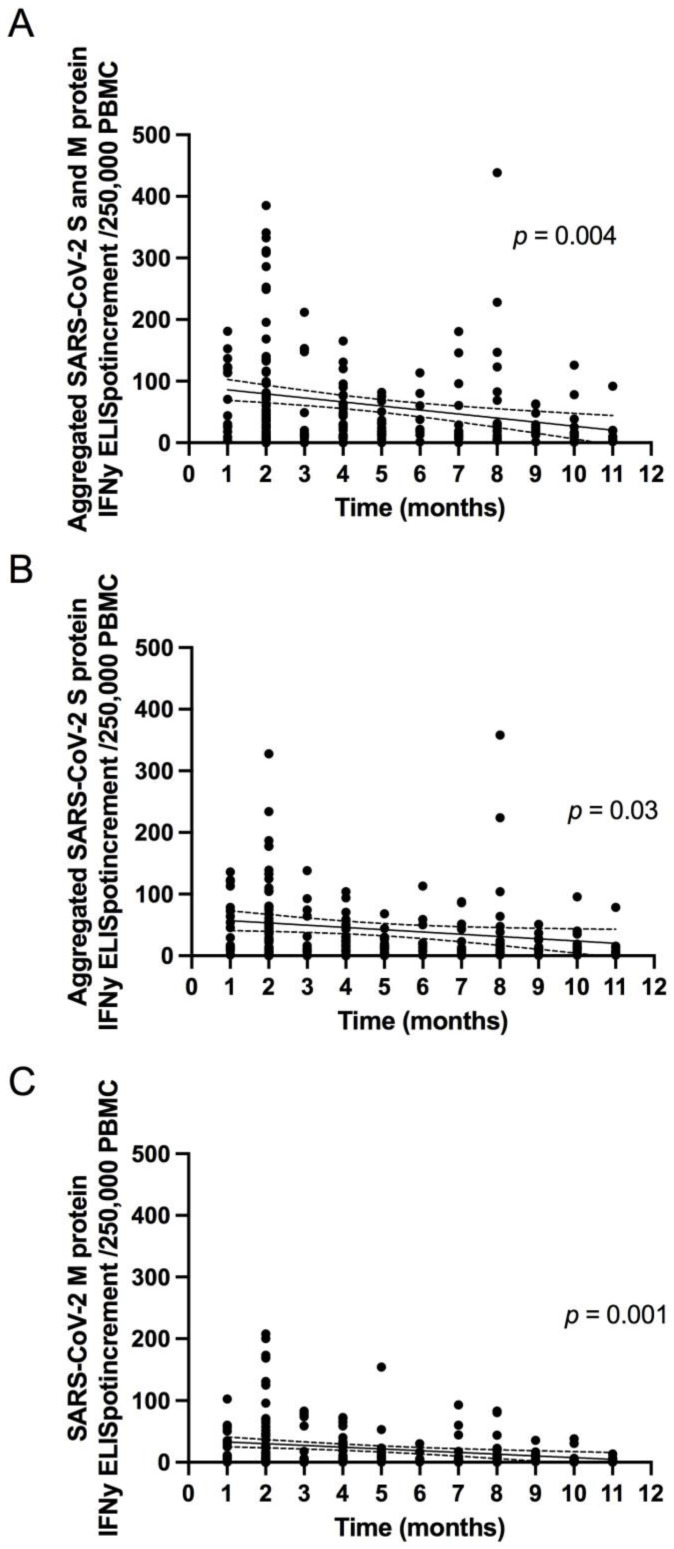
Correlation between aggregated number of SARS-CoV-2 Spike (S) and Membrane (M) protein specific T cell responses ((**A**); *p* = 0.004, R = −0.2), aggregated number of S protein specific T cell responses ((**B**); *p* = 0.03, R = −0.1) and M protein specific T cell responses ((**C**); *p* = 0.001, R = −0.2) and the time since initial COVID-19. Regression line (black line) with 95% confidence interval (dashed lines).

The aggregated number of SARS-CoV-2 specific T cell responses in the entire population decreased with increases in time since primary infection (Figure 5A). Similar results were obtained when the S and M peptide pools were analyzed separately (Figure 5B,C).

**Figure 6:**
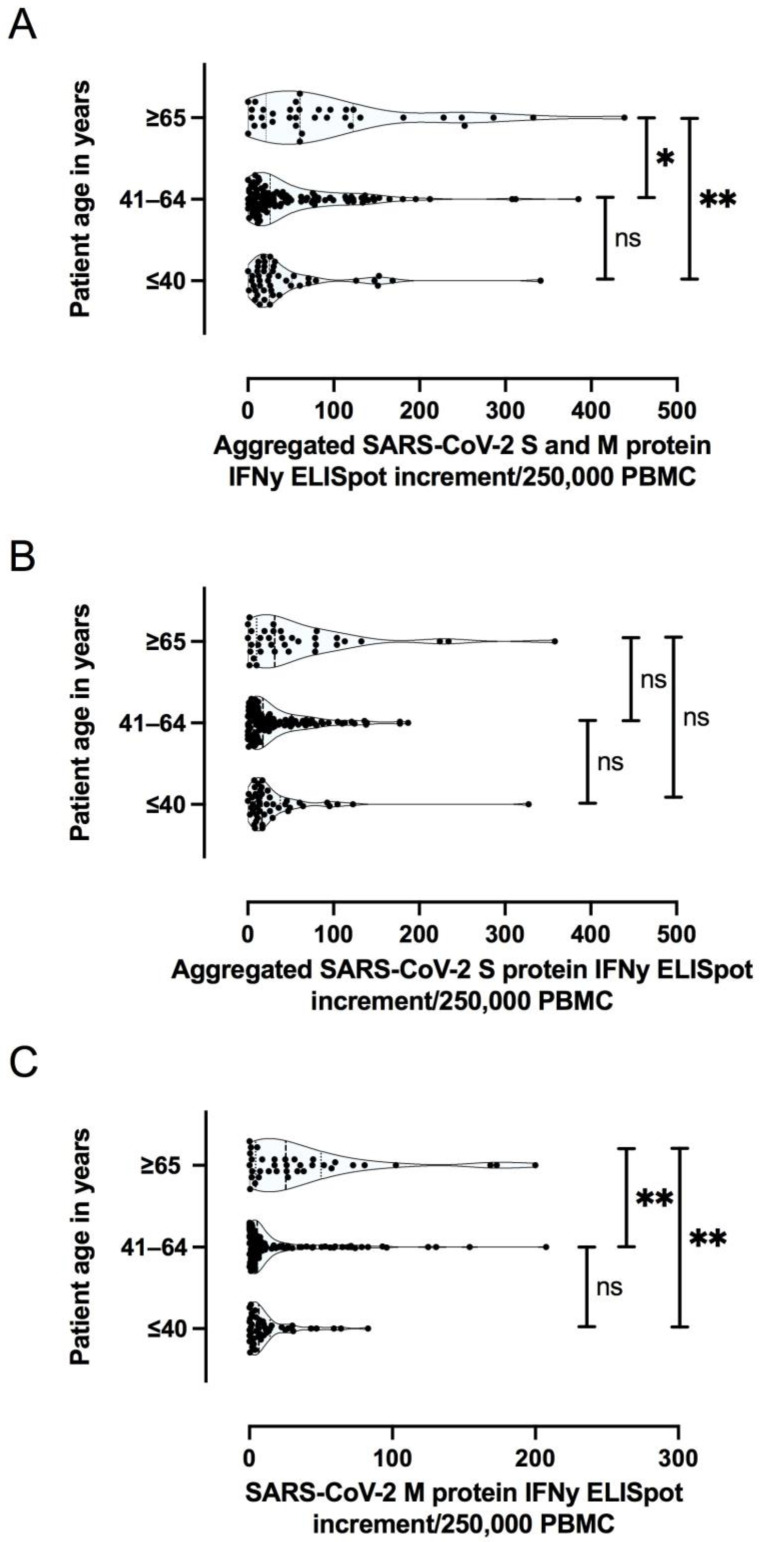
Aggregated IFNy ELISpot increment for SARS-CoV-2 Spike (S) and Membrane (M) protein specific T cell responses (**A**), aggregated number of S protein specific T cell responses (**B**), and M protein specific T cell responses (**C**), with respect to patient age. ** *p* < 0.005; * *p* < 0.05; ns not significant.

Furthermore, older patient age proved to be predictive of a stronger Membrane (M) protein specific T cell response, as detected by ELISpot assay; whereas there was no significant difference for Spike (S) protein with respect to patient age (Figure 6A–C).

In addition to patient age, univariate analyses showed that sex, body mass index, presence of anti-SARS-CoV-2 IgG antibodies, and disease severity were factors for the magnitude of aggregated Spike (S) and Membrane (M) protein specific T cell responses. However, multivariate analysis determined that only initial COVID-19 severity (*p* = 0.043; 95%CI, 0.74–46.96) and patient age (*p* = 0.031; 95%CI, 0.1–2.1) were independent predictors of strong T cell responses as shown by ELISpot assays, whereas there was a borderline association with time after primary infection (*p* = 0.068; 95% CI, −9.12–0.33) (Table 3).

The S-protein and M-protein specific T-cell responses were considered individually. Multivariate analysis determined that initial COVID-19 severity affected the S protein specific T cell responses, whereas age and time after primary infection affected the M protein specific T cell response (Table 4 and Table 5).

### 3.3. Specific Antibody Responses to SARS-CoV-2

#### 3.3.1. Specific Antibody Responses to SARS-CoV-2 Correlates with Neutralizing Antibodies in Addition to SARS-CoV-2 Specific T Cell Responses

**Figure 7:**
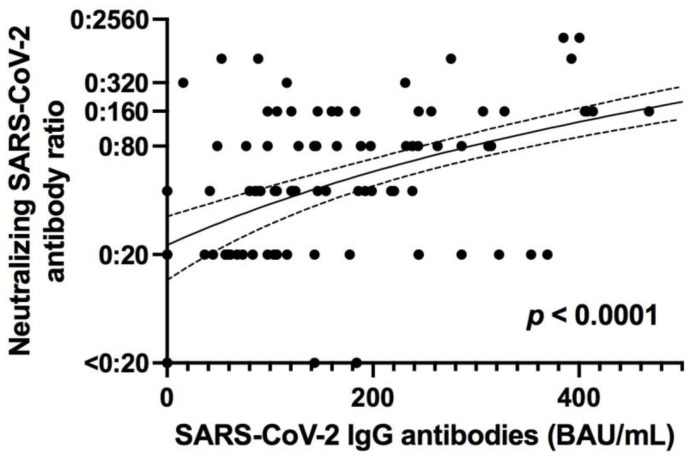
Correlation of neutralizing SARS-CoV-2 antibody ratio and SARS-CoV-2 IgG antibodies against S protein, as measured with a chemiluminescence enzyme immunoassay (CLIA). BAU, binding antibody unit; IgG, immunoglobulin G. *p <* 0.0001, R = 0.6, regression line (black line) with 95% confidence interval (dashed lines).

SARS-CoV-2 IgG levels were undetectable at the time of screening for twenty of our patients (10%); in contrast to SARS-CoV-2 specific T cell immunity, only one of these patients was receiving immunosuppressive therapy. Neutralizing anti-SARS-CoV-2 antibodies were evaluated in 176 out of 200 patients. Twelve out of 176 patients (6.8%) exhibited no neutralizing antibodies against SARS-CoV-2 (and no SARS-CoV-2 IgG antibodies). We found a statistically significant positive correlation between SARS-CoV-2 IgG levels (measured with CLIA) and levels of neutralizing antibodies against SARS-CoV-2 (*p* < 0.0001, R = 0.6; Figure 7).

**Figure 8:**
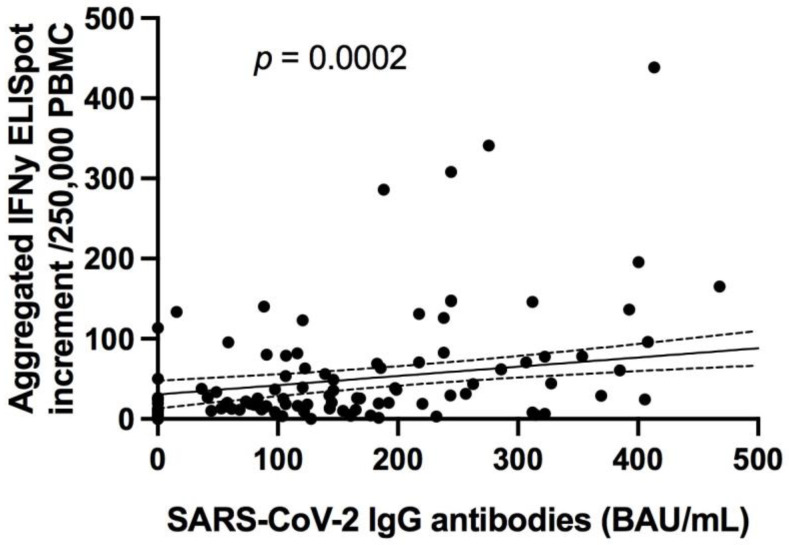
Correlation of SARS-CoV-2 IgG antibodies against S proteins and aggregated SARS-CoV-2 specific T cell responses in the ELISpot assays. *p =* 0.0002; R = 0.3, regression line (black line) with 95% confidence interval (dashed lines).

Of note, there was a significant correlation between the SARS-CoV-2 antibody titers and the magnitude of aggregated SARS-CoV-2 specific T cell responses in the ELISpot assay (*p <* 0.0001; R = 0.3; Figure 8).

#### 3.3.2. Specific Antibody Responses to SARS-CoV-2 Are Predominantly Influenced by the Severity of the Primary COVID-19

**Figure 9:**
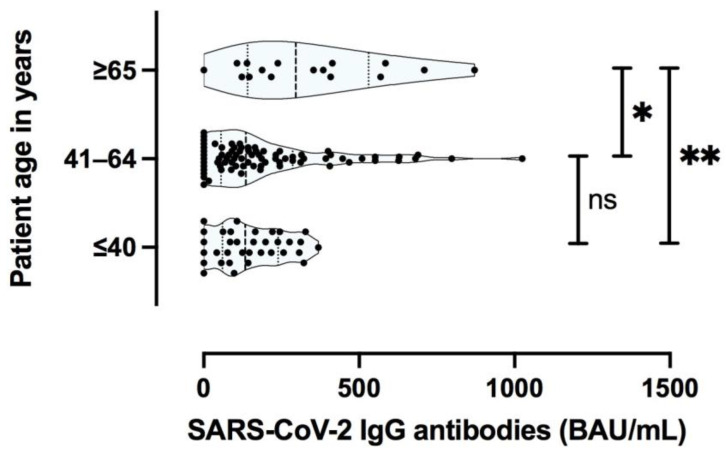
SARS-CoV-2 immunoglobulin G (IgG) antibodies by age (years). ** *p <* 0.005; *, *p <* 0.05; ns, not statistically significant.

Consistent with the T cell specific immunity results, advanced patient age was found to be a factor for higher SARS-CoV-2 specific anti S protein IgG titer levels (Figure 9).

In addition to the patient age, univariate analyses determined that patient sex, presence of SARS-CoV-2 specific T cells, and initial disease severity were prognostic factors for the magnitude of SARS-CoV-2 IgG titer levels. However, regarding SARS-CoV-2 specific IgG levels, multivariate analysis showed that initial COVID-19 severity (*p* < 0.0001; 95%CI, 63.8–163.8) and the presence of SARS-CoV-2 specific T cells were the only independent prognostic factor. There was a borderline association with patient age (Table 6).

## 4. Discussion

In a cohort of 200 unvaccinated COVID-19 convalescents, SARS-CoV-2 specific T cell- and humoral immunity was assessed up to almost one year after resolution of acute infection. The combined results of our study not only provide a comprehensive characterization of the immune responses after COVID-19 but also demonstrate that the severity of the initial disease predominantly affects cellular and humoral immunity against the Spike (S) protein even months after recovery from SARS-CoV-2 infection. Regarding Membrane (M) protein specific T cell immunity, patient age and time since SARS-CoV-2 infection were prognostic factors for long-term immunity. In addition, immunosuppression was an independent factor for the absence of T cell immunity. In our cohort, significant risk factors for hospitalization were older age, obesity, and male sex which is consistent with the findings of other studies [15,16,17].

Knowledge of the extent and quality of the long-term immune system response to SARS-CoV-2 is crucial for further risk assessment regarding the ongoing pandemic. In addition, understanding the natural history of immunity can help estimate the duration of immunity after vaccination. Factors that trigger initial and long-term individual immunity still remain to be characterized. T cell specific responses are initially detected in almost all SARS-CoV-2 infections [18,19] and have been associated with control of primary SARS-CoV-2 infection [19]. In our cohort, the main factor impairing the formation of T cell immune responses was an immunocompromised status of the patient. Thus, SARS-CoV-2-specific T cell responses (against the Spike (S) and Membrane (M) protein) were found significantly less frequently in immunocompromised patients, who are considered particularly at risk of severe forms of COVID-19 [20,21]. Consistent with our data, low T cell responses (and seroconversion rates) were observed in studies of various immunocompromised patient cohorts both during the natural course of SARS-CoV-2 infection and after vaccination [12,22,23,24,25,26]. However, because IFNγ ELISpot assays against three different viral Spike (S) peptide pools/proteins and one Membrane (M) protein pool provide the only read-out regarding T cell responses in our study, our findings may not provide comprehensive information about the complex overall T cell responses after primary SARS-CoV-2 infection [24]. Few studies have focused on long-term T cell immunity after COVID-19 [27,28,29]. Our results are consistent with those studies finding that T cell responses are detectable at least 6 months after initial infection, not only in symptomatic but also in asymptomatic subjects [28].

Virus-specific antibodies develop in most patients infected with SARS-CoV-2 within 5 to 15 days of infection [19,30], and the S protein is the target of neutralizing SARS-CoV-2 antibodies [13]. Although the number of studies of the persistence of SARS-CoV-2-specific immunity is increasing, data on long-term immunity after SARS-CoV-2 infection are still scarce. In addition, most of these studies focus on the persistence of circulating anti-SARS-CoV-2 IgG antibodies. These studies demonstrate that seroconversion is maintained over several months, as seen in our cohort [30,31]. Classification of patients according to the severity of initial COVID-19 showed that those who had a SARS-CoV-2 infection with more severe clinical manifestations had a stronger Spike (S) protein specific T cell and antibody response. This finding is consistent with results from other groups showing stronger SARS-CoV-2 specific T cell responses in patients who had recovered from severe COVID-19, as well as from other coronavirus infections, such as SARS and MERS, in which patients with more severe symptoms have higher levels of specific memory T cells against the virus [2,32,33]. Similar to the stronger T cell response observed in patients with acute COVID-19, stronger long-term T cell responses probably reflect a more severe course of disease with a stronger immunogenic environment created by higher viral load and inflammatory bystander activation [34,35]. In addition, neutralizing antibody titers and specific anti-S protein total antibody titers have been found to correlate with the severity of COVID-19 in our cohort and others [19,34,36].

In addition to COVID-19 severity, patient age and concomitant diseases have been shown to affect SARS-CoV-2 specific cellular immunity during the acute phase of the disease [37]. Typically, immune senescence predisposes older patients to infection and may hinder the development of protective immunity after immunization. Although the sample size in our study was limited, older participants showed a stronger T cell specific and antibody-specific immune response after COVID-19. Importantly, the age of the patient was an independent factor for the magnitude of T cell response (especially against the Membrane protein) and antibody responses, independent of the initial severity of COVID-19. This finding is in line with one previous study showing that even in advanced age, an immunological memory against SARS-CoV-2 was developed and was still present in most patients months after recovery [27,38,39,40,41]. Another potential explanation may be that older patients may had more repetitive exposures to seasonal coronaviruses that can induce cross-reactive T cells and antibodies recognizing regions of the Membrane (M) or Spike (S) proteins, which are conserved among several coronaviruses [42,43]. Especially, the Membrane (M) protein accounting for the overall shape of the viral envelope is the most abundant structural protein of the coronavirus family. Studies have shown that different coronavirus M proteins share the same overall basic structural characteristics and are most subject to evolutionary constraints [44,45,46]. A recent study of SARS-CoV-2 sequences confirmed this finding, showing that missense mutations in the Membrane protein gene were relatively uncommon [47]. Studies have shown that the Membrane protein can elicit similar immune responses as the S protein, making it a potential candidate for vaccines [48,49,50].

The current study has some limitations, mainly because longitudinal data for individual subjects are missing. In addition, all patients in this study survived their SARS-CoV-2 infection and thus were more likely to develop virus-specific immunity than patients who died as consequence of infection [37]. Nevertheless, this current prospective cross-sectional study describes the long-term persistence of cellular and humoral immunity to SARS-CoV-2, which is likely to be present for several months in the vast majority of adults after COVID-19. These characteristics are encouraging with respect to the longevity of cellular immunity to this novel virus and likely contribute to the relatively low rates of re-infection observed to date.

## 5. Conclusions

Consistent with the findings related to acute COVID-19, disease severity and patient age also influence immunity in the long-term.

## Figures and Tables

**Table 1:** Characteristics of the total patient cohort and the three subgroups: Patients were classified according to the most severe COVID-19 category according to the World Health Organization (WHO) classification, asymptomatic (B), moderate (C), severe disease (D). Comparison between the age and the body mass index (BMI) of the patients was performed with the Kruskal–Wallis test; comparison of sex distribution between groups was performed with the Chi-square test for trend. Statistical significance was set at the level of *p* < 0.05.

Variable	Total Group (A)	Asymptomatic Disease (B)	Moderate Disease (C)	Severe Disease (D)	*p* Value
No. patients (%)	200 (100)	116 (58)	63 (31.5)	21 (10.5)	
Median age, years (range)	50 (19–87)	48 (19–79)	56 (24–87)	56 (25–77)	<0.0001
Sex, men/women (%)	104/96 (52/48)	49/67 (42.2/57.8)	37/26 (58.7/41.3)	18/3 (85.7/14.3)	0.001
Median BMI (range)	26.5 (12.1–49.4)	25.8 (12.1–45.2)	27.5 (19.5–49.4)	27.8 (20.5–40.8)	0.017

**Table 2:** Univariate analysis and multivariate analysis of factors associated with absence of SARS-CoV-2 Spike (S) protein and Membrane (M) protein specific T cell response in ELISpot analysis. * significant.

	Univariate Analysis		Multivariate Analysis	
Variable	Odds Ratio (95% Confidence Interval)	*p* Value	Odds Ratio (95% Confidence Interval)	*p* Value
Immunosuppressive condition	7.42 (1.23–44.65)	0.029 *	12.53 (1.38–113.95)	0.025 *
Anti-SARS-CoV-2 IgG antibodies positive	0.056 (0.017–0.186)	<0.0001 *	0.052 (0.014–0.183)	<0.0001 *

**Table 3:** Univariate analysis and multivariate analysis of factors associated with aggregated Spike (S) protein and Membrane (M) protein SARS-CoV-2 specific T cell response in ELISpot analysis. * significant.

	Univariate Analysis		Multivariate Analysis	
Variable	Odds Ratio (95% Confidence Interval)	*p* Value	Odds Ratio (95% Confidence Interval)	*p* Value
Age, years	1.4 (0.5–2.4)	0.002 *	1.1 (0.1–2.0)	0.031 *
Sex (men/women)	24.2 (−2.5–51.0)	0.076	10.3 (−17.3–37.9)	0.46
Body mass index (kg/m²)	1.9 (−0.1–3.9)	0.056	1.1 (−0.9–3.1)	0.29
Anti-SARS-CoV-2 IgG antibodies positive	55.2 (10.25–100.1)	0.016 *	31.0 (−12.9–74.9)	0.17
COVID-19 severity	38.9 (19.4–58.4)	<0.0001 *	23.85 (0.74–46.96)	0.043 *
Time after SARS-CoV-2 infection (months)	−6.4 (−10.9–−2.08)	0.004 *	−4.4 (−9.2–0.33)	0.068

**Table 4:** Univariate analysis and multivariate analysis of factors associated with aggregated Spike (S) SARS-CoV-2 specific T cell response in ELISpot analysis. * significant.

	Univariate Analysis		Multivariate Analysis	
Variable	Odds Ratio (95% Confidence Interval)	*p* Value	Odds Ratio (95% Confidence Interval)	*p* Value
Age, years	0.8 (0.1–1.5)	0.03 *	0.4 (−0.3–1.2)	0.27
Sex (men/women)	16.1 (−4.0–36.1)	0.1	4.3 (−16.6–25.1)	0.7
Body mass index (kg/m²)	1.4 (−0.1–2.8)	0.07	0.7 (−0.8–2.2)	0.4
Anti-SARS-CoV-2 IgG antibodies positive	37.3 (3.6–71.0)	0.03 *	22.0 (−11.2–55.3)	0.19
COVID-19 severity	31.3 (19.4–58.4)	<0.0001 *	26.4 (8.9–43.8)	0.003 *
Time after SARS-CoV-2 infection (months)	−3.7 (−6.9–−0.4)	0.03 *	−1.6 (−5.2–1.9)	0.4

**Table 5:** Univariate analysis and multivariate analysis of factors associated with Membrane (M) protein SARS-CoV-2 specific T cell response in ELISpot analysis. * significant.

	Univariate Analysis		Multivariate Analysis	
Variable	Odds Ratio (95% Confidence Interval)	*p* Value	Odds Ratio (95% Confidence Interval)	*p* Value
Age, years	0.7 (0.3–1.0)	<0.0001 *	0.7 (0.3–1.0)	<0.0001 *
Sex (men/women)	8.4 (−1.7–18.5)	0.1	6.0 (−4.4–16.4)	0.26
Body mass index (kg/m²)	0.6 (−0.2–1.3)	0.13	0.4 (−0.4–1.2)	0.29
Anti-SARS-CoV-2 IgG antibodies positive	18.0 (1.0–35.0)	0.04 *	9.0 (−7.5–25.6)	0.28
COVID-19 severity	7.9 (0.4–15.3)	0.04 *	−2.5 (−11.2–6.2)	0.6
Time after SARS-CoV-2 infection (months)	−2.8 (−4.5–1.2)	0.001 *	−2.8 (−4.5–1.0)	0.003 *

**Table 6:** Univariate analysis and multivariate analysis of factors associated with SARS-CoV-2 specific anti S protein IgG titer levels. * significant.

	Univariate Analysis		Multivariate Analysis	
Variable	Odds Ratio (95% Confidence Interval)	*p* Value	Odds Ratio (95% Confidence Interval)	*p* Value
Age, years	4.1 (1.7–6.5)	0.001 *	2.3 (−0.02–4.6)	0.052
Sex (men/women)	73.9 (3.8–143.9)	0.039 *	20.6 (−42.5–83.7)	0.52
Body mass index (kg/m²)	4.9 (−0.2–10.0)	0.059	3.7 (−0.7–8.2)	0.09
SARS-CoV-2 specific T cells present	168.9 (45.6–292.2)	0.008 *	157.3 (48.7–265.8)	0.005 *
COVID-19 severity	141.8 (93.6–190.2)	<0.0001 *	113.8 (63.8–163.8)	<0.0001 *
Time after SARS-CoV-2 infection (months)	−5.2 (−16.7–6.4)	0.3		

## Data Availability

The raw data supporting the conclusions of this article will be made available by the authors upon request.
